# A Novel CalB-Type Lipase Discovered by Fungal Genomes Mining

**DOI:** 10.1371/journal.pone.0124882

**Published:** 2015-04-21

**Authors:** Maria E. Vaquero, Laura I. de Eugenio, Maria J. Martínez, Jorge Barriuso

**Affiliations:** Centro de Investigaciones Biológicas, Consejo Superior de Investigaciones Científicas, Ramiro de Maeztu 9, 28040 Madrid, Spain; The University of Hong Kong, HONG KONG

## Abstract

The fungus *Pseudozyma antarctica* produces a lipase (CalB) with broad substrate specificity, stability, high regio- and enantio-selectivity. It is active in non-aqueous organic solvents and at elevated temperatures. Hence, CalB is a robust biocatalyst for chemical conversions on an industrial scale. Here we report the *in silico* mining of public metagenomes and fungal genomes to discover novel lipases with high homology to CalB. The candidates were selected taking into account homology and conserved motifs criteria, as well as, phylogeny and 3D model analyses. The most promising candidate (PlicB) presented interesting structural properties. PlicB was expressed in a heterologous host, purified and partially characterized. Further experiments will allow finding novel catalytic properties with biotechnological interest.

## Introduction

Carboxylester hydrolases (E.C. 3.1.1) are a group of enzymes which includes: esterases (E.C.3.1.1.1) defined by their ability of hydrolyze carboxyl esters from short chain acylglycerol and lipases (triacylglycerol lipase, E.C. 3.1.1.3) enzymes which are capable of releasing long-chain fatty acids from water-insoluble carboxylic esters[1]. Both enzymes are able to catalyze esterification and transesterification reactions in presence of organic solvents. The distinction between lipases and esterases is not clear. Several unsuccessful attempts aimed at differentiating “lipases” from “esterases” by using criteria such as primary sequence comparisons, structural features, and kinetic parameters have been analyzed[2].A single feature is not sufficient to differentiate esterases from lipases, and therefore, a new bio-physico-chemical classification of lipolytic enzymes was proposed [3].

These closely related enzymes are distributed among animals, plants and microorganism. One of the most important lipases used in biotechnological applications is CalBfrom the basidiomycete *Pseudozyma antarctica*,formerly known as *Candida antarctica* [4].This yeast belongs to the *Ustilagomycetes* class, which includes the plant pathogen *Ustilago maydis*, where few lipases have been recently characterized [5, 6].CalB shows broad substrate specificity, stability, and high regio-and enantio-selectivity. Moreover, itis active under conditions that are unnatural to most other enzymeslike in non-aqueous organic solvents and at elevated temperatures[7]. Hence, CalB is a robust biocatalyst for chemical conversions on an industrial scale [8].

Structurally, CalB belongs to the α/β hydrolase foldfamily, represented by a conserved core structure composed ofseven central β strands, flanked on both sides by ten α-helices [7].The fold presents a stable scaffold for the catalytic triad composed by Ser105, His224 and Asp 187, in nearly all lipases the residues around the nucleophileserine are highly conserved presenting the motif GXSXG; exceptions to this pattern are: TWSQG in CalB and AHSMG in lipase from *Saccharomyces cerevisiae*[9]. CalB presents a potential lid formed by helix α5, however, in contrast with other lipases, does not shows interfacial activation [10].

Advances in DNA sequencinghave allowed studyingthe genomes of an enormous number of organisms in a short time. As an example, the Joint Genome Institute (JGI) from the US department of energy (DOE) has more than 300 fungal genomes completed andavailable in its website (http://www.jgi.doe.gov/). Furthermore, the study of DNA directly sequenced from environmental samples it is known as metagenomics. There are many metagenomes sequencing projects deposited in public databases, such as MG-RAST, IMG/M, and CAMERA [11]. Bioinformatics approaches to analyze these datasets allow the finding of new enzymes in the available DNA sequences [12].

In this work, we report the *in silico* mining of public fungal genomes to discover lipaseswith high homology to CalB. The candidates were selected taking into account homology and conserved motifs criteria, as well as, phylogeny and 3D modelanalyses. The most promising candidate was expressed in heterologous hosts, purified and its catalytic properties studied.

## Materials and Methods

### Screening and selection of candidate

To identify the conserved motifs in the sequencesof the *P*.*antarctica*lipase B family (abH37.01) all sequences available in “The Lipase Engineering Database” [9] from this family were downloaded (http://www.led.uni-stuttgart.de/), and subjected to analysis using MEME software (http://meme.sdsc.edu/meme/intro.html). The only conserved amino acids sequence detected in all cases was WSQG, comprising the catalytic Ser from CalB [7, 9].

To look for the putative lipases containing the conserved motifs WSQG in the fungal genomes and environmental metagenomes, automatically predicted proteins from all 300 fungal genomes at the JGI web-site containing the terms “esterase” (26089 sequences) or “lipase” (16855 sequences) were downloaded using the Advanced Search option. Also, eighty-one nucleotide datasets from different metagenomes, selected from diverse environments to maximize genetic variability, were downloaded from the databases MG-RAST (http://metagenomics.anl.gov/) and IMG/M (http://img.jgi.doe.gov) (7 million assembled contigs, Barriuso and Martínez, 2014), and translated in the six possible reading frames to aminoacidic sequences. From the total pool of sequences, the ones contained the conserved motifs WSQG were selected using the Bioedit 7.1.3 software (32 hits).Alternatively, as a second screening strategy, protein similarity searches were performedat the JGI and the NCBI-NR databases using BLASTp algorithm (e value 10^–2^) using CalB lipase as a query (29 hits).

### Sequence and phylogenetic analysis

Candidate sequences were compared by means of BLASTp against the “The Lipase Engineering Data Project” (LED) databases; those belonging to the CalB-family were subjected to a phylogenetic analysis. Selected candidates were aligned using MUSCLE, and an un-rooted tree was created using Maximum-likelihood methods with a boostrap of 2000(MEGA6 software).

### Structure modeling

Selected sequences were aligned versus CalB using ClustalW. Putative signal peptides were predicted using SignalP 4.0. N-glycosylation sites and disulphide bonds were predicted using NetNGlyc1.0 server and DiANNA 1.1 web server respectively. The three-dimensional structures of candidates were modeled on the SWISS-MODEL server in the automatic mode. The modelswere exhaustively analyzed using PyMol 1.1.

### Cloning procedures and heterologous expression

DNA coding sequences for the mature protein of putative lipase gene from the *Plicaturopsis crispa* genome (JGI protein ID 57710) was codon optimized for its expression in *Escherichia coli* and *Komagataella* (= *Pichia*) *pastoris* and synthesized by ATG:biosynthetics (Merzhausen, Germany). The gene was cloned into the vectors pET28a(+) (Merck, Darmstadt, Germany), using *Nco*I and *Not*I sites, under the transcriptional control of the T7 promoter for its expression in *E*. *coli*. The pET28:PlicB construct was transformed into *E*. *coli* BL21(DE3)pLysS cells. Expression and solubility was checked by SDS-PAGE gels, using lysates from single colonies grown at 37°Cin LB with kanamycin (50μg/mL) and chloramphenicol (34μg/mL)until optical density at 600 nm reached 0.6. Then, the cultures were induced with IPTG (1mM), grown at 37°C or 16°C for 16h, and harvested by centrifugation (6000 x g, 20 min, 4°C). Cell pellets were resuspended in 5 mL lysis buffer (10 mMTris-HCl, pH 7), sonicated (Misonix S4000; Qsonica, Newtown, CT, USA), separated from the supernatant fraction, and checked for protein solubility using 10% polyacrylamide gels.

For expression in *P*. *pastoris*, *plicB* gene was cloned into pPIC9 vector (Invitrogen, Calrsbad, CA, USA) under the transcriptional control of the methanol-inducible *AOX*1 promoter, using *EcoR*I and *Not*I sites. The pPIC9:PlicBconstruct was transformed into *P*. *pastoris* KM71 and GS115 strainsas described before [13].Activity screening was performed using single colonies from the transformation plates grown in 20 mL YEPS medium in 100 mL flasks with 0.5% w/v of methanol to induce gene expression. The flasks were incubated at 28°C and 250rpm and 0.5% w/v methanol was added daily for maintaining the induction. The esterase activity was checked using the lipase activity assay kit II (Sigma-Aldrich, Steinheim, Germany).

### PlicB production, purification and characterization

Positive clones from *P*. *pastoris*, secreting PlicB were selected for protein production and purification. One liter flasks with 100 mL of YEPS medium, inoculated with 3,5 mL of overnight YPD cultures (DO_600_ 8–10), were incubated at 28°C and 250 rpm. When maximum activity was reached (4 days), the cells were harvested by centrifugation (6000 ×*g* at 4°C) and the supernatant concentrated in 10000 MWCO Amicon-Ultra Centrifugal filters (Merck-Millipore, Darmstadt, Germany). Supernatant was equilibrated with 0.5M ammonium sulfate in 20mMTris-HCl pH 7 buffer and applied to a Octyl-Sepharose cartridge (GE Healthcare Life Sciences, Uppsala, Sweden). Proteins were eluted with a linear decreasing gradient (0.5-0M) of ammonium sulfate in the same buffer. Fractions containing lipase activity were concentrated, dialyzed and subjected to size exclusion chromatography in a HiLoad 16/600 Superdex 75 pg (GE Healthcare) column equilibrated and eluted with 20mM Tris-HCl pH 7 buffer containing 0.15M NaCl. Dextran blue (2000 kDa), Aprotinin (6,5kDa), Ribonuclease A (13,7 kDa), Carbonic anhidrase (29 kDa), Ovoalbumin (44 kDa) and Conalbumin (75 kDa), (Calibration kits; GE Healthcare), were used as standards to calibrate the column in order to evaluate PlicB quaternary structure.

The apparent molecular weight of the purified PlicB was estimated from SDS-PAGE. Precision Plus Protein Dual Color Standards (Bio-Rad, Hércules, CA, USA) were used in running 10% polyacrylamide gels.N-linked-carbohydrate content (%) was estimated as the difference between the apparent molecular mass of the protein before and after deglycosylation withEndoglycosidase H (Roche, Mannheim, Germany). For N-deglycosylation, the purified protein was dialyzed against sodium citrate buffer, pH 5.5, and then incubated with Endo H at 37°C for 24 h, following the manufacturer’s instructions. Protein concentration was determined by theBradford (Bio-Rad), bovine serum albumin was used as standard [6].

### Peptide mass fingerprinting using MALDI-TOF mass spectrometry

The purified protein was analyzed by SDS-PAGE in a 10% polyacrylamide gel and stained with SYPRO Ruby (Bio-Rad). The band was excised and subjected to tryptic in-gel digestion in a DigestPro MS digestor (Intavis, Köln, Germany). MS analyses of the tryptic peptides were performed in an Autoflex III MALDI-TOF/TOF mass spectrometer (Bruker Daltonics, Bremen, Germany) controlled by the flexControl3.0 software (Bruker Daltonics). Three of the tryptic peptides were chosen to carry out fragmentation and sequencing. MALDI-MS and MS/MS data were combined through the BioTools 3.0 program (Bruker Daltonics) to search against the non-redundant protein database from the NCBI using the MASCOT 2.3 search engine (Matrix Science, London, UK). Scores greater than 75 were considered significant (p<0.05).

### Esterase and lipase activity assays

PlicB activity against triglycerides was tested in tributyrin agarose plates (1% agarose, 1% glyceryl tributyratepreviously homogenized in 20 mMTris-HCl pH 7 buffer). Twenty-five micrograms of purified protein were dropped onto the plates, followed by incubation at 28°C overnight. Alternately, plates with Tween20/40/80 substrates were used (1% agarose, 0.2% Tween 20/40/80and 0.6mM CaCl_2_).Fifty micrograms of purified protein were dropped onto the plates followed by incubation at 28°C for 24-48h. Fatty acids released from substrates produced aturbid halo.On the other hand, PlicB lipase activity was quantified spectrophotometrically using the Lipase activity assay kit II (Sigma-Aldrich), and compared with that from commercial non immobilized CalB (lyophilized powder; Sigma-Aldrich).

The hydrolysis of *p*-nitrophenyl butyrate (*p*NPB), *p*-nitrophenyllaurate (*p*NPL) and *p*-nitrophenylpalmitate (*p*NPP) were assayed in presence of 1% (v/v) Genapol X-100 as surfactant, as described previously [13], using an Shimadzu UV-1800 spectrophotometer with magnetic stirring (600 rpm) and temperature control at 25°C. One unit of activity (1U) is defined as the amount of enzyme releasing 1 μmol of *p*-nitrophenol (ε410 = 15,200 M-1cm-1) per minute under the defined conditions.

### pH and temperature stability

To determine the enzymes stability at different pHs, the enzyme were incubated in 10 mM Britton-Robinson buffer from pH 4 to 9 at 4°C. Samples were taken at 24h to calculate residual activity using the *p*NPB assay as describe above. The initial activity of the enzyme was taken as 100%.Thermal stability was determined by the temperature at which 50% of activity was lost in 10 min incubation (T50 value) the protein was incubated in 20 mMTris-HCl pH 7 buffer at a range from 30 to 60°C, cooled on ice, and rewarmed to room temperature for 5 min prior to residual activity determination by Lipase activity assay kit II (Sigma-Aldrich).

## Results and Discussion

### Genomes and metagenomes screening

After the search of conserved sequence WSQG in 81 environmental metagenomes and putative proteins annotated as lipase and/or esterase in more than 300 fungal genomes, only 21 and 11 candidates contained the motif, respectively. Moreover, analysis and comparison of these sequences in “The Lipase Engineering Database” retrieved no *C*. *antarctica* lipase B family members (abH37.01). In the case of candidates from metagenomes all belonged to bacteria; as previously reported the abundance of fungal sequences in this data sets it is very low [11].

**Fig 1:**
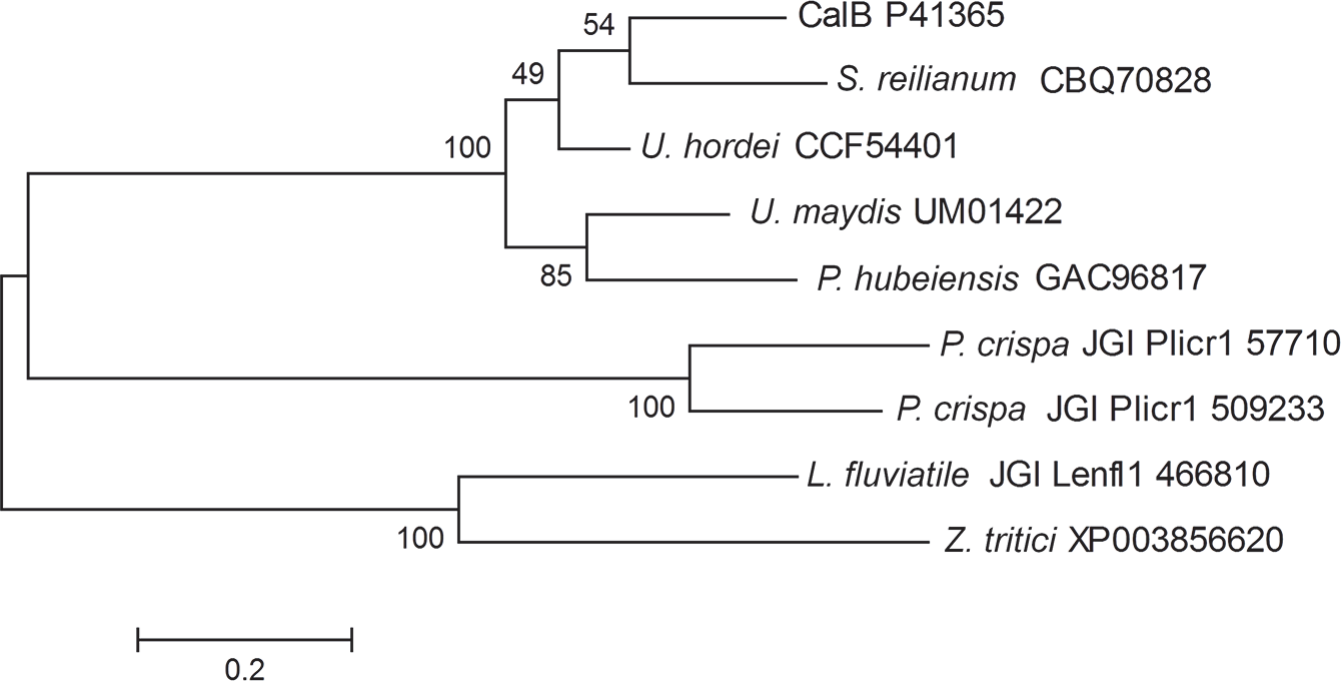
Phylogenetic analysisof CalB and eight potential CalB-like lipase candidates. The tree was built using MEGA6 software, boostrapvalues (2000replicates) are shown next to the branches. The name of the specie is followed by the sequence accession number.

Alternatively, a second strategy based on sequence homology search against JGI and NCBInr databases using CalB as query rendered 29 sequences. After a deeper analysis of these sequences,8 belonged to the abH37.01 family and were subjected to a phylogenetic study (Fig 1).

Interestingly, candidates from *P*. *crispa* and *Lentithecium fluviatile* only ariseusing the second search strategy. This is due to absence of the conserved WSQG motif in *P*. *crispa* sequences, and to the annotation of *L*.*fluviatile* sequence as anα/β-hydrolase instead of “lipase” or “esterase” in the database. In this sense it is worthy to mention the convenience of applying different search strategies to avoid the loss ofpotential candidates.

Lipase sequences from *Ustilago* and *Sporisorium*, Gramineae parasites closely related and commonly known as smut fungi [14], grouped in the same branch than CalBand lipases from the anamorphic yeast-like fungus *Pseudozyma*,that also belongs to the *Ustilaginales* [15].Several of these lipases are protected by international patents due to its biotechnological potential interest [16], and lipase B from *U*. *maydis* (Uml2) has been recently characterized [6]. In a second cluster with a higher phylogenetic distance from CalB, grouped the putative lipases from the *Dothideomycetes*: *Zymoseptoriatritici* (ID XP003856620), a wheat pathogen[17] and *Lentithecium fluviatile* (JGI/Lenfl1/ID 466810), from fresh water habitats.

Finally,lipases from the Agarical *Plicaturopsiscrispa* (JGI/Plicr1/ID 57710 and 509233) grouped in a different branch at an intermediate phylogenetic distance between *Ustilaginales* and *Dothideomycetes*. Putative protein ID 509233 seems to be a truncated form of the putative protein ID 57710 presenting 70 amino acids less, probably due to a wrong automatic genome annotation. *P*. *crispa* is a white rot basidiomycete, and is the first member of the Amylocorticiales order to be sequenced [18]. This fungus is an effective decayer, colonizing predominantly dead branches of deciduous trees[17].

In this kind of fungi,secreted lipases may play a role for nutrition and/or damage of host cells to help penetrate host tissues. This enzymes may be implicated in the initial degradation of the epicuticular waxes and cuticle, which consist of a mixture of long-chain fatty acids, aldehydes, alkanes, primary and secondary alcohols, ketones and wax esters [19].

### Sequence analysis and structural model

**Fig 2:**
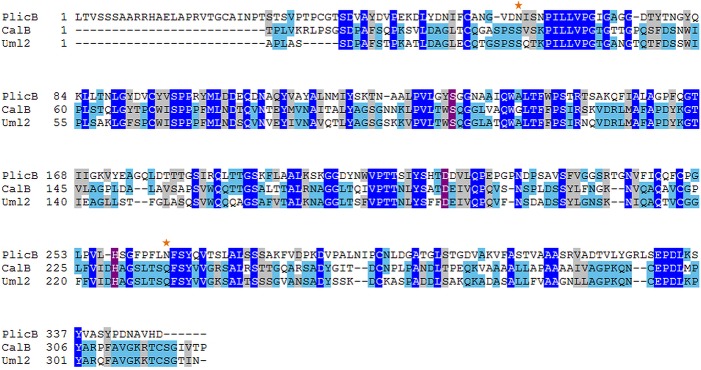
Protein sequences alignment of the mature regions of PlicB, CalB and Uml2. Residues completely conserved at a given position are indicated in white on blue. Putative catalytic residues are highlighted in white on purple. And potential N-glycosylation sites are marked by a star.

The putative lipase sequence PlicB from basidiomycete *P*. *crispa* (protein ID 57710) was selected as the most promising candidate, since it was very similar to the characterized enzymes CalB and Uml2, but still presented some interesting differences. PlicB possess 367 amino acids with a theoretical mass of 37k Da and contains a predicted signal sequence of 19 amino acids. Mature PlicB presented 30% sequence identity and 44% similarity against CalB(Fig 2). Molecular models of the selected candidate were generated using SWISS-MODEL server in the automatic mode. Thetemplatechosenin all cases was the structure of CalB (PDB: 4k6h). The 3D model from PlicB adjusted with a Qmean value of -5.25 (S1 Fig).

Residues Ser122, Asp218 and His257 are probably responsible for the catalytic activity of the protein as they superpose in the model with the catalytic triad of CalB. It is worth to notice that PlicB was the only candidate found that did not presented the conserved motif WSQG forming the shoulder of the catalytic Ser. Instead of a tryptophan PlicB presented a tyrosine preceding the catalytic serine (Fig 2), similar to cutinases from *Fusarium solani* and *Aspergillus oryza*e[9]. The tryptophan in CalB seems not to be crucial as it is shown in the mutant W104A,which presents an expanded pocket with increase activity against alcohols [20].

Analyzing potential disulphide bonds, PlicB possesses two(Cys54-Cys94 and Cys-250-Cys293), one less than CalB and Uml2[6]. The overall structure of PlicB (S1 Fig) is a globular α/β-type protein with a central β-sheet composed of seven β-strands flanked by α-helices. CalB has a semi-covered active site consisting of α5 (as the lid) and α10 (as activation element), forming a narrow hydrophobic channel with the catalytic residues inside[21]. In PlicB model α5 is replaced by a loopforming a cleft-like enzymatic cavity.

### PlicB production, purification and characterization

**Fig 3:**
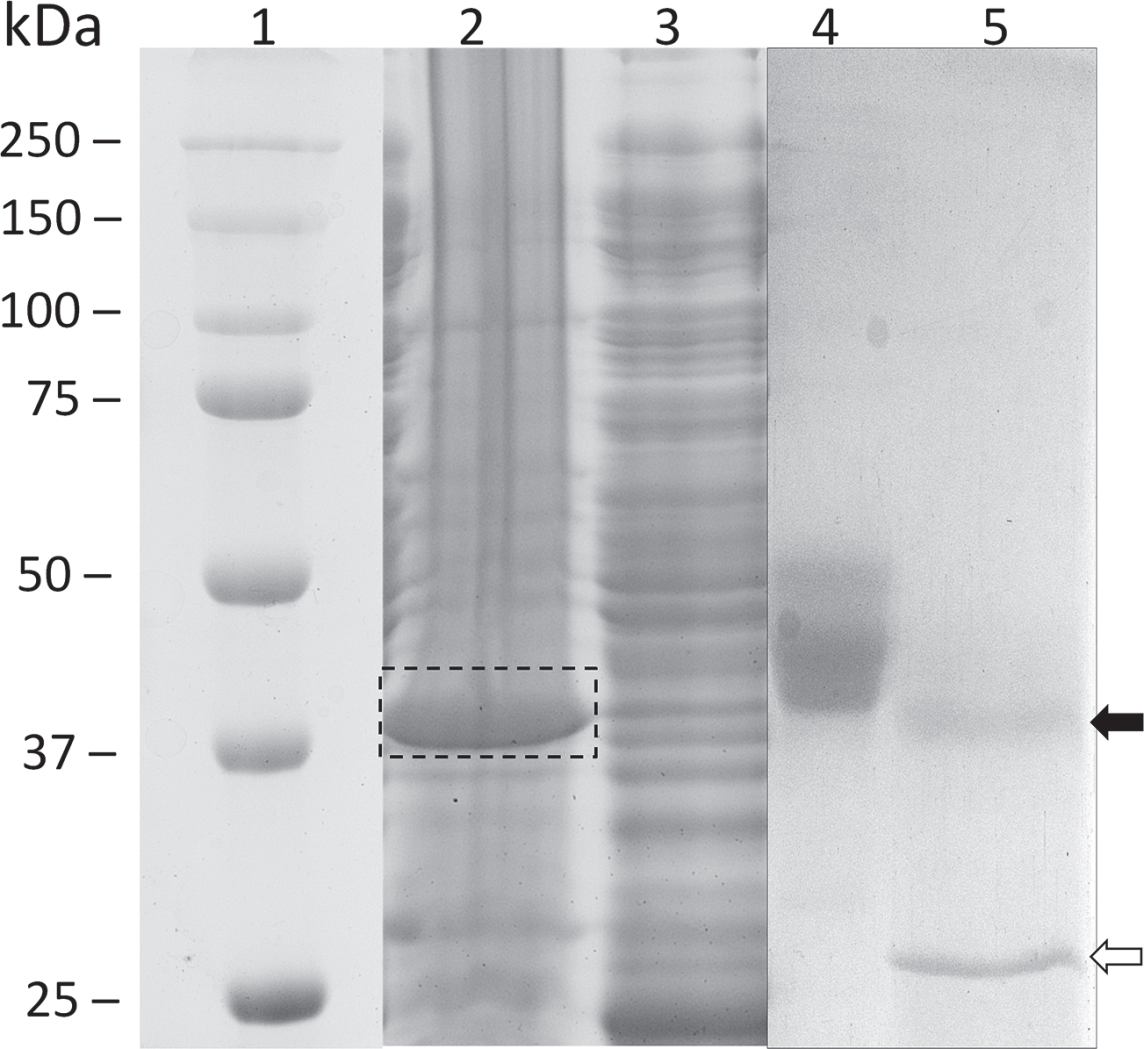
SDS-PAGE of PlicB expressed in *E*. *coli* and *P*. *pastoris*. Lane 1, molecular mass markers; lane 2–3 pellet and supernatant respectively, of crude extract from PlicB expressed in *E*.*coli* BL21(DE3)pLysS grown at 37°C. The dashed box indicates the insoluble expression of PlicB. Lane 4, purified PlicB expressed in *P*. *pastoris* GS115. Lane 5, purified PlicB expressed in *P*. *pastoris* after Endoglycosidase H treatment. The migration of deglycosylated PlicB (black arrow) and Endoglycosidase H (open arrow) are indicated.

Taking into account sequence and phylogenetic analysis, as well as the3D molecular models, protein from *P*.*crispa*ID 57710was selected for heterologous expression. The mature sequence of *plicB* gene was expressed in *E*. *coli* and *P*. *pastoris*. In the first case, the protein obtainedwas insoluble, inactive and located inside inclusion bodies (Fig 3). The deposition of unfolded or partially misfolded protein is a common problem in this host, especially when expressing eukaryotic proteins [22]. On the other hand, *P*. *pastoris* resulted to be an optimum heterologous hostfor this kind of enzymes, as previously described [23]. Different *P*. *pastoris* strains were tested, and the best producer clone was obtainedfrom strain GS115.Purification of PlicB was performed from methanol-induced cultures grown for 96h: culture supernatant was applied onto hydrophobic and size exclusion chromatography columns, yielded 4mg/L of purified protein of an estimated molecular mass of 48kDa (Fig 3), although its theoretical molecular mass was 37 kDa. After enzymatic deglycosylation of the protein by Endoglycosidase H treatment, a unique band of approximately 37kDa appeared as seen by SDS-PAGE, indicating a 20% of sugars N-liked (Fig 3), in accordance with the two N-glycosylationsites predicted (Asn60 and Asn264).The molecular weight of PlicB calculated by size exclusion chromatography using a HiLoad 16/600 Superdex 75 pg column was 47.8 kDa, very similar to that obtained under denaturing conditions in SDS-PAGE, which would indicate that PlicB is a monomer in the conditions tested, according to CalB previous results [24]. PlicB identity was confirmed by mass fingerprinting analysis (data not shown).

Lipase activity of purified enzyme was checked ontributyrinand Tween 20/40/80 plates(S2 Fig). Tween 20/40/80 are polysorbate detergents containing esters of fatty acids with different chain length: lauric acid, palmitic acid and oleic acid, respectively. Although the purified protein was able to hydrolyze all the substrates assayed, PlicB produced a more visiblehalo in Tween 40 compared to Tween 20 after overnight incubation, while the halo in Tween 80 plates appeared only after 48h incubation. This ability has already been shown in CalB and Uml2[6]. PlicB lipase activity was quantified and compared to CalB by using a spectrophotometric approach (Lipase assay kit II, Sigma-Aldrich)(Table 1).By this method, PlicB showed 4-fold higher lipase activity compared to CalB. Esterase activity against *p*-nitrophenyl (*p*NP) esters of different fatty acids length chain (Table 1), revealed that PlicB presented higher activity towards short chain substrates as *p*-NPB (C4), than towards fatty acid esters of 12 and 16 carbons of acyl chain. Although CalB presented 43-fold higher activity than PlicB against *p*NPB and *p*NPL, hydrolysis of long chain *p*NPP by the two lipases was similar. Furthermore, Buerth et al. [6] compared the specific activity of Uml2 and CalB against *p*NP-esters in reactions containing DMSO (5% v/v) and 5mM deoxycholate. CalB resulted around 10 times more active towards *p*NPB and *p*NPL than Uml2, while the activity was similar against *p*NPP.

**Table 1:** Specific activity of ofPlicB and commercial non-immobilized CalB.

Substrate	Fattyacids	Specificactivity (mU mg-1)	
		PlicB	CalB
*p*NPB	C4:0	100 ± 19	4300 ± 500
*p*NPL	C12:0	32 ± 2	209 ± 5
*p*NPP	C16:0	22 ± 4	15 ± 8
Lipase kit	C4:0	1400 ± 106	343 ± 6

Esterase activity was assayed usingdifferent acyl long chain *p*NP esters as substrate (1.5mM in presence of 1% Genapol X-100). Lipase activity was assayed using the lipase assay kit II (Sigma-Aldrich).

Inspection of the molecular surfaces of PlicB model and CalB revealed notable structural differences in the substrate binding pocket. CalB showed a funnel-like binding site able to accommodate large substrates [25, 26], while PlicB showed a cleft-like enzymatic cavity. Moreover, it has been suggested that α5 helix in CalB possessed flexibility and interacts with the acyl part of a substrate[27]; in the case of PlicB this α5 helix it is not present. Marked differences in lid regions among CalB homologues have previously been described. Substitution of CalB lid with that from relatedlipasesresulted in chimeric proteins with altered catalytic properties and enantioselectivity[27]. These facts could contribute to the different substrate recognition and hydrolytic efficiency of PlicB.

In terms of regioselectivity toward the position of the acyl group of triglycerides, lipases can be classified into *sn*-1,3-regiospecific (*Rhizomucormiehei* lipase), *sn*-2 specific (*C*. *antarctica*lipase A) and nonspecific lipases (*C*. *rugosa* lipase). The substrate utilized in the Lipase activity assay kit II (Sigma-Aldrich) is a thioester analog of tributyrin (2,3 dimercapto-1-propanoltributyrate, DMPTB)[28]. Thus, *sn*1,3-specific lipase can hydrolyze only one of the DMPTB-thioester groups, whereas nonspecific lipases couldhydrolyze two groups [29]. According to that, the differences in lipase activity with this substrate could be related with the fact that PlicB may be classified as nonspecific lipase while CalB would be an 1,3-specific lipase.

**Fig 4:**
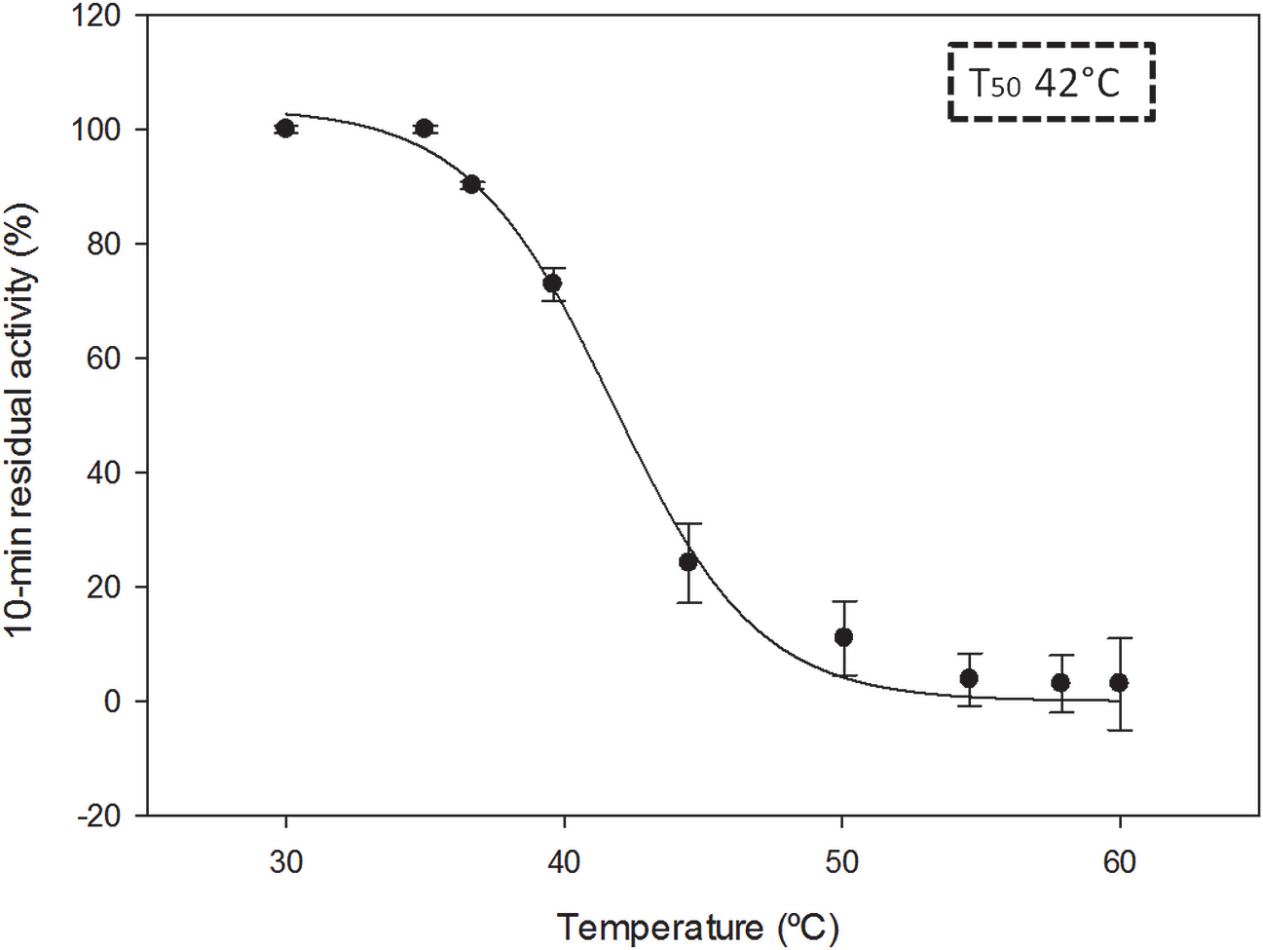
Temperature stability. Purified PlicB in 20mM Tris-HCl pH 7 was incubated 10 min at different temperatures (30–60°C). Temperature stability is presented as T_50_ value (dashed box).

In spite of the substrate specificity differences found, the temperature stability (T_50_) (Fig 4)and pH stability of PlicB (42°C and stable between pH 6 and 9) revealed a thermal and pH stability situated in the same range of CalB(46°C) and other CalB-like lipases [30]. Enzymes stable at wide range of pH and temperature are desirable for their biotechnological application[1].

In summary, genome mining has been proved as useful strategiesin order to discover new enzyme with potential improved properties. Putative lipases from the CalB family were screened using two different strategies. Most of theselected candidates corresponded with previously characterized proteins, however, a new putative lipase (PlicB), lacking the conserved motif (WSQG) in the catalytic Ser shoulder, was identified. PlicB was expressed in two different heterologous hosts, purified, and partly characterized.

## Supporting Information

S1 Fig Comparison of PlicB 3D model and the molecular structure of CalB. A) PlicB structural model, ribbon diagram (upper part) and molecular surface (lower part). B) CalB structure (PDB: 1tca) ribbon diagram (upper part) and molecular surface (lower part). Catalytic serine is represented in magenta. The alpha helix delimiting the enzyme´s lid region in CalB is represented in red, while PlicB model presents a loop (in pink). PlicB posses a cleft-like enzymatic cavity, while CalB posses a funnel-like binding site (lower part).
(PDF)

S2 Fig PlicBlipase activity tested in Tributyrin and Tween 20/40/60 plates. Twenty-five micrograms and 50 μg were dropped into Tributyrin and Tween20/40/60 plates respectively and incubated at 28°C for 24 h, in the case of Tween60 the incubation was for 48 hours.
(PDF)
