# Supplementary material for: A Novel CalB-Type Lipase Discovered by Fungal Genomes Mining
Source: PLoS One. 2015 Apr 21;10(4):e0124882. doi: 10.1371/journal.pone.0124882 (PMC4405274; doi:10.1371/journal.pone.0124882)
Supplement: S1 Fig — A) PlicB structural model, ribbon diagram (upper part) and molecular surface (lower part). B) CalB structure (PDB: 1tca) ribbon diagram (upper part) and molecular surface (lower part). Catalytic serine is represented in magenta. The alpha helix delimiting the enzyme´s lid region in CalB is represented in red, while PlicB model presents a loop (in pink). PlicB posses a cleft-like enzymatic cavity, while CalB posses a funnel-like binding site (lower part). (PDF) [file pone.0124882.s001.pdf]

S1 Fig. Comparison of PlicB 3D model and the molecular structure of CalB.

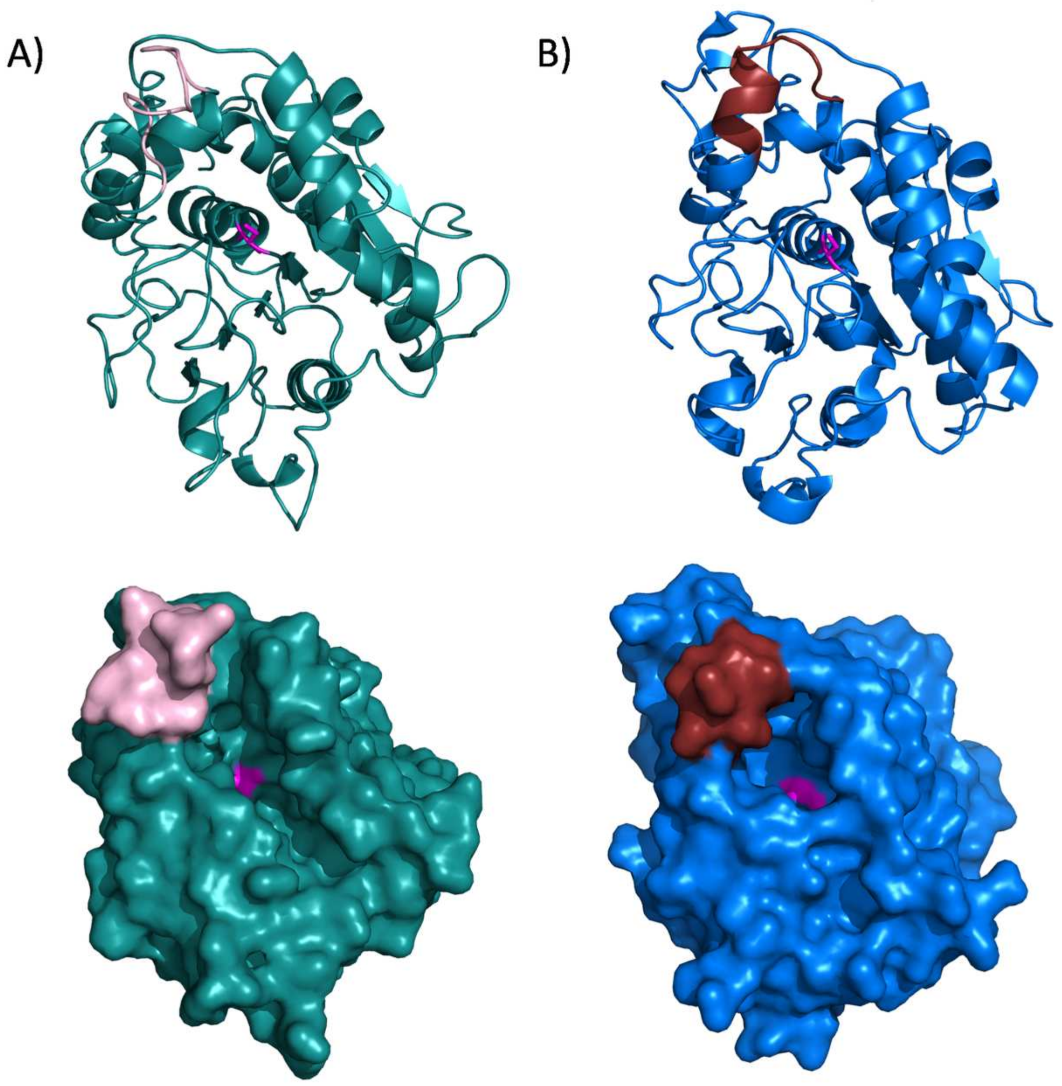

A) PlicB structural model, ribbon diagram (upper part) and molecular surface (lower part). B) CalB structure (PDB: 1tca) ribbon diagram (upper part) and molecular surface (lower part). Catalytic serine is represented in magenta. The alpha helix delimiting the enzyme´s lid region in CalB is represented in red, while PlicB model presents a loop (in pink). PlicB posses a cleft-like enzymatic cavity, while CalB posses a funnel-like binding site (lower part).
