# Supplementary material for: A Novel CalB-Type Lipase Discovered by Fungal Genomes Mining
Source: PLoS One. 2015 Apr 21;10(4):e0124882. doi: 10.1371/journal.pone.0124882 (PMC4405274; doi:10.1371/journal.pone.0124882)
Supplement: S2 Fig — Twenty-five micrograms and 50 μg were dropped into Tributyrin and Tween20/40/60 plates respectively and incubated at 28°C for 24 h, in the case of Tween60 the incubation was for 48 hours. (PDF) [file pone.0124882.s002.pdf]

S2 Fig. PlicB Lipase activity tested in Tributyrin and Tween 20/40/60 plates.

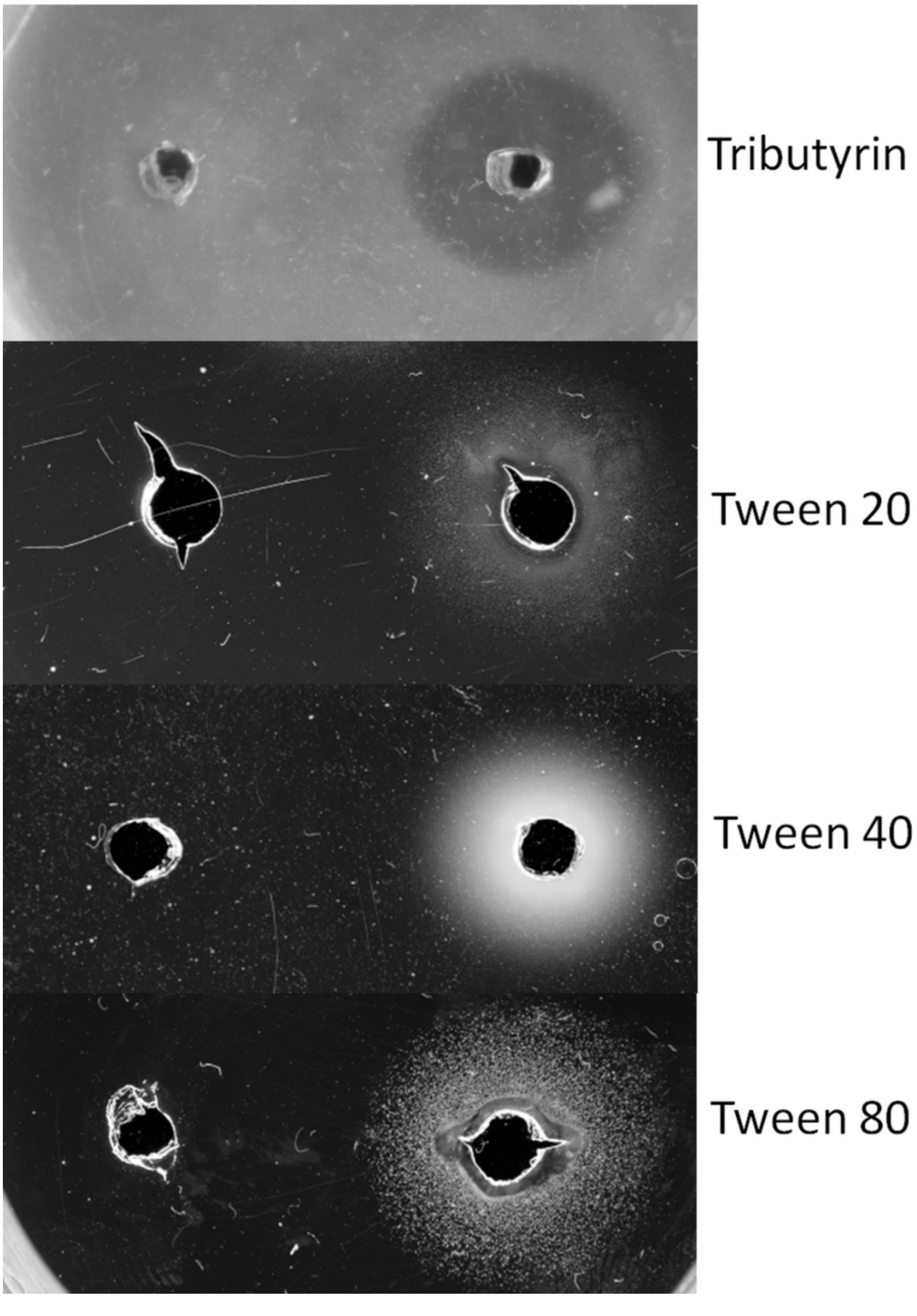

Twenty-five micrograms and 50 μg were dropped into Tributyrin and Tween 20/40/60 plates respectively and incubated at 28 °C for 24 h, in the case of Tween 60 the incubation was for 48 hours.
